# Trajectories and predictors of HIV care retention among individuals receiving ART in rural South Africa: a group-based trajectory modelling analysis

**DOI:** 10.7189/jogh.15.04187

**Published:** 2025-07-01

**Authors:** Aimée Julien, Jean Juste Harrisson Bashingwa, Annelies Van Rie, Mi-Suk Kang Dufour, Nkosinathi Masilela, Rebecca L West, Dumisani Rebombo, Francesc Xavier Gómez-Olivé, Kathleen Kahn, Sheri A Lippman, Audrey Pettifor, Chodziwadziwa Whiteson Kabudula

**Affiliations:** 1 Department of Family Medicine and Population Health, University of Antwerp, Antwerp, Belgium; 2 SAMRC/Wits Rural Public Health and Health Transitions Research Unit (Agincourt), School of Public Health, Faculty of Health Sciences, University of the Witwatersrand, Johannesburg, South Africa; 3 Division of Biostatistics, University of California Berkeley, Berkeley, USA; 4 School of Public Health, Boston University, Boston, USA; 5 Sonke Gender Justice, Cape Town, South Africa; 6 Department of Medicine, Center for AIDS Prevention Studies, University of California, San Francisco, USA; 7 Department of Epidemiology, University of North Carolina, Chapel Hill, USA; 8 Centre de recherche du Centre Hospitalier Universitaire Sainte-Justine, Montréal, Canada

## Abstract

**Background:**

Successful retention in care of people living with HIV remains a challenge and a cornerstone of ending the epidemic. A better understanding of retention predictors could guide an evidence-based approach to target interventions. We sought to characterise HIV care retention trajectories among individuals receiving antiretroviral therapy (ART) in a rural South Africa setting, and to determine factors associated with those trajectories.

**Methods:**

We conducted a population-based cohort study of individuals receiving ART in ten health care facilities within the Agincourt Health and Socio-Demographic Surveillance System site in Mpumalanga, South Africa, in 2015–18. We used group-based trajectory modelling to identify clusters of individuals with similar retention trajectories and assessed the association between socio-demographic factors and trajectory groups using multinomial logistic regression.

**Results:**

Among 1689 individuals receiving ART during the study period, five distinct trajectory groups were identified: 30.8% had gradually decreasing retention over time, 10.2% had late increasing retention, 20.7% had early increasing retention, 7.8% had early decreasing retention, and 30.5% had consistently high retention. Individuals in the consistently high retention group were more likely to be female and aged ≥40 years. In contrast, those in the early decreasing retention group were more likely to be male, aged <30 years, and with temporary resident status. Individuals in the early increasing retention group were more likely to be from villages included in a HIV Treatment as Prevention community mobilisation study. Education, marital status, and socioeconomic status were not significantly associated with group membership. Months on ART were weakly associated with group membership.

**Conclusions:**

Five distinct retention trajectories were observed and associated with specific sociodemographic factors. Our study offers a data-driven approach to inform the design of targeted interventions to improve HIV care retention. Interventions and policies addressing socioeconomic and system-level factors are essential to achieving better outcomes in high-burden areas.

South Africa has one of the largest HIV epidemics in the world, with an estimated 7.6 million people living with HIV, and an HIV prevalence of 17.8% among adults (15–49 years) [1]. South Africa also has the largest HIV care and antiretroviral therapy (ART) program in the world. In 2016, the universal test-and-treat strategy was implemented in South Africa to help achieve the Joint United Nations Programme on HIV/AIDS (UNAIDS) 90-90-90 targets by 2020 and 95-95-95 targets by 2030 [2-5]. By 2022, an estimated 95% of people living with HIV knew their status, but only 75% were on ART, and 60% were virally suppressed [1].

These estimates should be interpreted with caution, as overreporting of testing and underreporting of HIV status, particularly among men, has also been reported in the region, highlighting the importance of accurate monitoring of population testing and progress towards the UNAIDS targets [6]. Nevertheless, studies in the region have shown that men are indeed tested, linked, and retained in HIV care at lower rates than women [7-10], and that important barriers to testing, linkage to, and retention in care remain [11-19]. Socioeconomic barriers include poverty, HIV-related stigma, fear of disclosure, lack of social support from family or partner, lack of support from health care system/providers, unequal gender norms, violence victimisation, cultural and religious beliefs, built environment conditions (*i.e.* inadequate home, schools, and/or recreational areas infrastructure; proximity to services, lack of transport), and mobility [11,13,16-26]. However, to our knowledge, little is known about specific patterns and trajectories of HIV care retention in Mpumalanga, South Africa, or about the factors associated with these trajectories that can inform the design of tailored and comprehensive interventions in the region.

While most people living with HIV do not follow a single, linear care trajectory or longitudinal pathways through the HIV care continuum, most studies and analyses of the HIV care continuum are cross-sectional. Consequently, these studies fail to elucidate the specific pathways and trajectories that people living with HIV follow and the factors associated with different trajectories [27,28]. In this study, we sought to characterise trajectories of HIV care retention over time among individuals receiving ART in a rural setting with high HIV prevalence. We also aimed to determine the factors associated with those trajectories to help inform the design of interventions to improve HIV care retention.

## METHODS

### Study setting

This study was a secondary analysis of the Tsima study conducted in the Agincourt Health and Socio-Demographic Surveillance System (HDSS) study area, which was established in 1992 by the South African Medical Research Council and Wits University Rural Public Health and Health Transitions Research Unit [29]. The Agincourt HDSS is in the Bushbuckridge sub-district of Mpumalanga Province, South Africa, approximately 500km northeast of Johannesburg. Mpumalanga is a predominantly rural province characterised by high levels of poverty, unemployment, and labour migration, as well as a high prevalence of HIV. In 2022, the estimated HIV prevalence in Mpumalanga was 20.8% among adults aged ≥15 years [30].

Through an annual household and vital events census update, the South African Medical Research Council /Wits-Agincourt research unit maintains a detailed database and sampling frame of over 20 000 households and about 117 000 resident individuals living in 31 fully enumerated villages. Of these, 15 villages participated in the Tsima study, a cluster-randomised trial conducted between 2015 and 2018 to evaluate a theory-based community mobilisation intervention addressing known social barriers to engagement in HIV care [13,31]. The intervention sought to increase HIV testing uptake, thereby decreasing undiagnosed infections, and to improve HIV care engagement among adults aged 18–49, to reduce new infections and improve health outcomes. To measure changes in HIV testing, linkage to HIV care, and retention in care, population-based HDSS longitudinal census data were linked to a clinic-based electronic tracking system (Agincourt HDSS-Clinic Link) deployed at the 10 public health care facilities serving the 15 study communities.

The Tsima study obtained written informed consent from patients to capture their clinical record data and conduct the census-clinic record linkage using deterministic and probabilistic approaches. Over 95% of adults aged ≥18 years accessing the ten study clinics during the Tsima study period (1 August 2015 to 31 July 2018) consented to linkage of their census and clinic data. Once informed consent was obtained from clinic attendees, they became study participants and their identifiers were captured in HDSS-Clinic Link by data capturers. These identifiers were then searched in the Agincourt HDSS database using an algorithm based on the Fellegi-Sunter probabilistic record linkage model. Potential matches were reviewed in the patient's presence to resolve any uncertainty about their identity. Key identifiers for posterior links with the Agincourt HDSS database used in the search (*i.e.* name, surname, age or date of birth, sex, village of residence, cell phone number, national ID, name of another person living in the household) were confirmed in previous research [32,33].

### Analytic study population

For this study’s analyses, we first created a record for every adult (≥18 years) resident of the 31 HDSS villages. We linked their clinical data to their census records, resulting in an open cohort of 71 369 individuals. Individuals were eligible for inclusion in this secondary analysis if they had started ART on or before 1 August 2015 (Tsima’s study start), had HIV care retention status information, and had complete census information linked to their clinic record (including socioeconomic status (SES), education, marital status, and resident status). We excluded individuals if they had started ART after 1 August 2015, if they had missing HIV care retention status information, or if they had missing census information in their record. By including only participants who started ART on or before 1 August 2015, we ensured that they would be followed for the entire duration of the Tsima study.

### Outcome and covariates

The study outcome was a time-varying binary variable describing whether an individual remained or not in ART care during the Tsima study period (1 August 2015 to 31 July 2018). We used the same definitions of HIV care retention as the Tsima parent study. Individuals were considered retained in care if they were on treatment or if patients did not experience a lapse in medication coverage for ≥90 days [13]. For the few people living with HIV who were not eligible for ART in the first year of the Tsima study, before implementation of universal test-and-treat in the region on 1 September 2016, we defined retention as having regular monitoring documented as a CD4 or viral load test every six months. Clinical services were assessed continuously, and for analytic purposes, the retention outcome was operationalised as a binary variable (retained or not retained) per three-month windows (quarters).

We extracted covariates, including gender, age, education, marital status, resident status, and SES, from the Agincourt HDSS longitudinal census data. SES is based on an absolute household wealth index computed from a list of household asset indicators. The wealth index is divided into household wealth quintiles, the first quintile represents the poorest households, and the fifth the richest households. A detailed description of the SES measure computation has been described elsewhere [34].

### Statistical analyses

We used *R*, version 4.3 (R CoreTeam, Vienna, Austria) for all analyses. We applied group-based trajectory modelling (GBTM) to estimate the HIV care retention trajectories of study participants. We used general finite mixture regression models. Finite mixture models are a widely used technique for modelling unobserved heterogeneity or approximating general distribution functions in a semi-parametric manner using the expectation-maximisation algorithm [35-38]. The mixture is assumed to comprise K components (groups), where each component follows a parametric distribution. In practical applications, the number of components is unknown and must be estimated by repeatedly running the algorithm using different starting values. Accordingly, the model was developed in two steps using the ‘flexmix’ package in the RStudio software [35,39]. First, we ran the model five times for K = 2, 3, …, 10 components, resulting in 45 runs. The best starting value was determined based on the number of components that minimised the Akaike information criterion (AIC), Bayesian information criterion (BIC), and integrated classification likelihood (ICL). Second, we used the information obtained from the first step and ran a mixture of binomial models using the same finite mixture regression technique described above. Given our binary study outcome (retained or not retained), we chose the binomial model. Lastly, we assigned each participant membership to a distinct trajectory group. For the model to provide well-separated trajectory groups (entropy near 1.0), the proportion of the sample in each trajectory should be at least 5% [38]. Furthermore, we used a multinomial logistic regression model to assess the association of sociodemographic characteristics (*i.e.* age, gender, education, marital status, resident status, and SES) with distinct group trajectories.

#### Use of GBTM *vs* other methods

Although GBTM is a finite mixture model, it is also a semi-parametric model for longitudinal data. It assumes a discrete population distribution and thus can distinguish subgroups or subclasses of homogeneous individuals with a similar trajectory. Unlike traditional growth mixture models, which can estimate the within-class variance, GBTM assumes no variation between individuals in the same class. This means that with GBTM, researchers can discern differences between subgroups but not differences within subgroups. GBTM also estimates fewer parameters, making it faster to run with fewer errors, which can lead to results that are easier to interpret because the model is less complex [38].

## RESULTS

### Cohort profile

**Figure 1:**
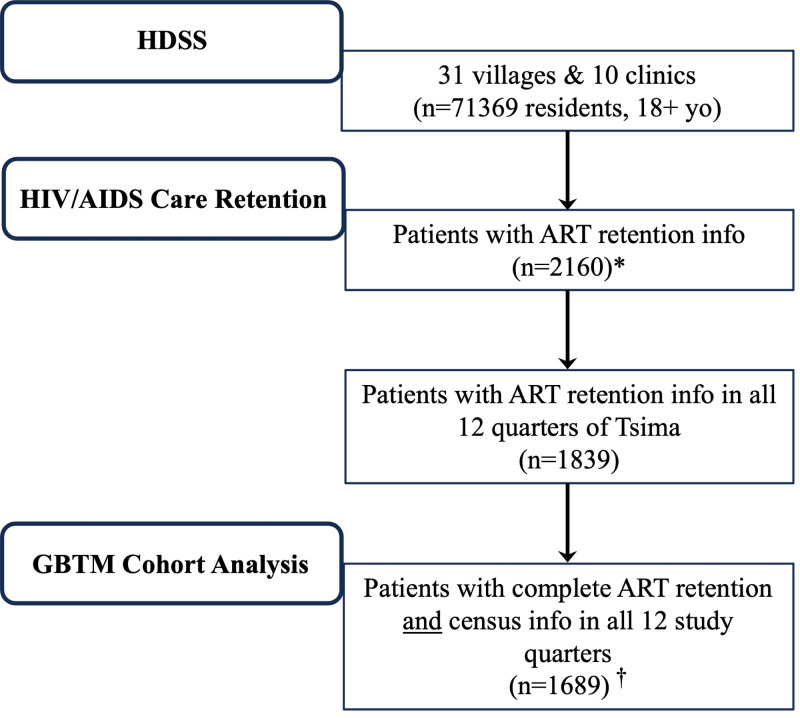
Flowchart of the study population. *Patients with HIV care or treatment visit during the Tsima study period. ^†^Complete cases without missing retention or census information, including SES, marital status, resident status, and education. ART – antiretroviral therapy, GBTM – group-based trajectory modelling, HDSS – Health and Socio-Demographic Surveillance System, SES – socioeconomic status, yo – years old.

Of the 71 369 residents in the 31 villages of the Agincourt HDSS, 2160 patients had an HIV care or treatment visit during the study period (**Figure 1**). Among these, 1839 patients had ART retention information in all 12 quarters of the study, 78.7% were female, 30.5% were on ART for ≤12 months, 45.5% were 40–49 years of age, 44.2% had a secondary education, 46.9% were single, 82% had permanent resident status in the study area, and 54.5% were in the intervention arm of the Tsima study (**Table 1**). Because 4% of the cohort had missing SES information, 2.2% had missing education information, and 5.9% had missing resident status information, we performed a complete analysis of the 1689 individuals with complete data. These patients were generally very similar to patients with complete covariate information concerning observed baseline characteristics; thus, bias in comparative samples is unlikely (Table S1 in the **Online Supplementary Document**).

**Table 1:** Characteristics of participants at baseline

	n (%)
**All participants**	1839
**Gender**	
Female	1448 (78.7)
Male	391 (21.3)
**Months on ART**	
0–12	561 (30.5)
13–24	261 (14.2)
25–36	274 (14.9)
37–48	258 (14.0)
49–60	175 (9.5)
≥61	310 (16.9)
**Age group**	
18–29	79 (4.3)
30–39	512 (27.8)
40–49	836 (45.5)
≥50	412 (22.4)
**SES quintiles**	
1	361 (19.6)
2	358 (19.5)
3	343 (18.7)
4	344 (18.7)
5	359 (19.5)
Missing	74 (4.0)
**Education**	
Matric and tertiary	592 (32.2)
Primary	394 (21.4)
Secondary	813 (44.2)
Missing	40 (2.2)
**Marital status**	
Married/cohabiting	610 (33.2)
Separated/divorced	256 (13.9)
Single	863 (46.9)
Widowed	110 (6.0)
**Resident status**	
Permanent	1508 (82.0)
Temporary	223 (12.1)
Missing	108 (5.9)
**Tsima study arm**	
Control	836 (45.5)
Intervention	1003 (54.5)

ART – antiretroviral therapy, SES – socioeconomic status

### HIV care retention trajectories

**Figure 2:**
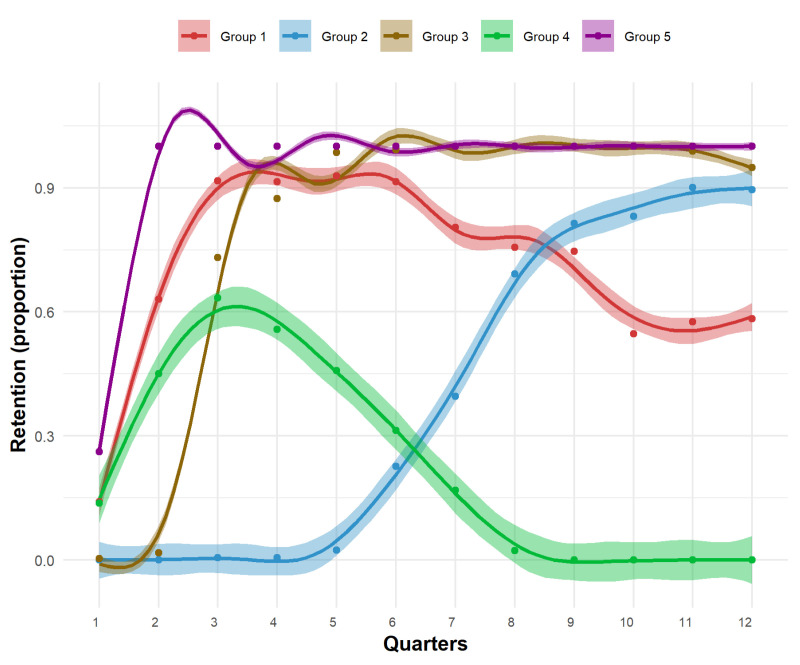
HIV care retention trajectory groups.

The selection of distinct trajectory groups was based on visual inspection of plots of posterior probabilities (rootograms), and the three quality criteria (BIC, AIC, and ICL). The BIC, AIC, and ICL values remained unchanged for k = 5, 6, 7, and 8; however, they improved for k = 10 (Figures S1 and S2, Table S2 in the **Online Supplementary Document**). Although increasing the number of groups yielded improvements in statistical fit (BIC, AIC, and ICL), based on the requirement of a minimum group size of at least 5% of the overall cohort, the five-group model was considered the best fit (**Figure 2**) [38]. The five distinct trajectories were: individuals with gradually decreasing retention over time (group 1) (30.8%), individuals whose retention increases late (after quarter 5 of Tsima) (group 2) (10.2%), individuals whose retention increased early (within the first 2 quarters of Tsima) (group 3) (20.7%), individuals with decreasing retention (group 4) (7.8%), and individuals with consistently high retention (group 5) (30.5%). Most participants were females (79.6%), had been on ART for ≤12 months (30.6%), aged 40–49 (45.6%), evenly distributed by SES, had a secondary education (45.6%), single (47.7%), and had permanent resident status in the study area (87.9%) (**Table 2**).

**Table 2:** Characteristics of study population by HIV care retention trajectory group*

	GBTM cohort	Group 1	Group 2	Group 3	Group 4	Group 5
**All participants**	1689	520 (30.8)	172 (10.2)	350 (20.7)	131 (7.8)	516 (30.5)
**Gender**						
Female	1344 (79.6)	412 (79.2)	124 (72.1)	296 (84.6)	91 (69.5)	421 (81.6)
Male	345 (20.4)	108 (20.8)	48 (27.9)	54 (15.4)	40 (30.5)	95 (18.4)
**Months on ART**						
0–12	516 (30.6)	152 (29.2)	58 (33.7)	105 (30.0)	56 (42.7)	145 (28.1)
13–24	236 (14.0)	80 (15.4)	19 (11.0)	51 (14.6)	20 (15.3)	66 (12.8)
25–36	249 (14.7)	65 (12.5)	23 (13.4)	57 (16.3)	23 (17.6)	81 (15.7)
37–48	238 (14.1)	72 (13.8)	25 (14.5)	61 (17.4)	9 (6.9)	71 (13.8)
49–60	165 (9.8)	62 (11.9)	14 (8.1)	27 (7.7)	8 (6.1)	54 (10.5)
≥61	285 (16.9)	89 (17.1)	33 (19.2)	49 (14.0)	15 (11.5)	99 (19.2)
**Age group**						
18–29	69 (4.1)	23 (4.4)	8 (4.7)	10 (2.9)	17 (13.0)	11 (2.1)
30–39	480 (28.4)	134 (25.8)	44 (25.6)	108 (30.9)	57 (43.5)	137 (26.6)
40–49	770 (45.6)	241 (46.3)	71 (41.3)	168 (48.0)	38 (29.0)	252 (48.8)
≥50	370 (21.9)	122 (23.5)	49 (28.5)	64 (18.3)	19 (14.5)	116 (22.5)
**SES quintiles**						
1	346 (20.5)	116 (22.3)	32 (18.6)	72 (20.6)	27 (20.6)	99 (19.2)
2	336 (19.9)	106 (20.4)	31 (18.0)	62 (17.7)	21 (16.0)	116 (22.5)
3	332 (19.7)	90 (17.3)	26 (15.1)	86 (24.6)	31 (23.7)	99 (19.2)
4	330 (19.5)	99 (19.0)	33 (19.2)	68 (19.4)	22 (16.8)	108 (20.9)
5	345 (20.4)	109 (21.0)	50 (29.1)	62 (17.7)	30 (22.9)	94 (18.2)
**Education**						
Primary	364 (21.6)	108 (20.8)	34 (19.8)	83 (23.7)	21 (16.0)	118 (22.9)
Secondary	770 (45.6)	229 (44.0)	76 (44.2)	168 (48.0)	68 (51.9)	229 (44.4)
Matric and tertiary	555 (32.9)	183 (35.2)	62 (36.0)	99 (28.3)	42 (32.1)	169 (32.8)
**Marital status**						
Single	806 (47.7)	241 (46.3)	77 (44.8)	170 (48.6)	75 (57.3)	243 (47.1)
Married/cohabiting	575 (34.0)	180 (34.6)	61 (35.5)	125 (35.7)	36 (27.5)	173 (33.5)
Widowed	104 (6.2)	42 (8.1)	15 (8.7)	20 (5.7)	6 (4.6)	21 (4.1)
Separated/divorced	204 (12.1)	57 (11.0)	19 (11.0)	35 (10.0)	14 (10.7)	79 (15.3)
**Resident status**						
Permanent	1484 (87.9)	457 (87.9)	148 (86.0)	310 (88.6)	107 (81.7)	462 (89.5)
Temporary	205 (12.1)	63 (12.1)	24 (14.0)	40 (11.4)	24 (18.3)	54 (10.5)
**Tsima study arm**						
Control	765 (45.3)	260 (50.0)	70 (40.7)	121 (34.6)	63 (48.1)	251 (48.6)
Intervention	924 (54.7)	260 (50.0)	102 (59.3)	229 (65.4)	68 (51.9)	265 (51.4)

ART – antiretroviral therapy, GBTM – group-based trajectory modelling, SES – socioeconomic status

*Presented as n (%) unless specified otherwise.

#### Factors associated with trajectory groups & probability of trajectory group membership

**Figure 3:**
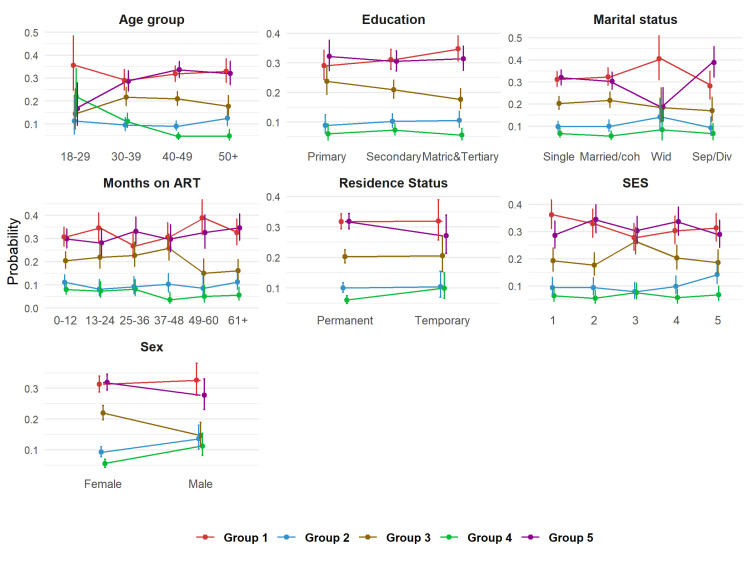
Predicted probabilities of trajectory group membership by retention predictor. ART – antiretroviral therapy, SES – socioeconomic status.

In bivariate analysis, socio-demographic characteristics differed by trajectory group (**Table 2**). Several factors were independently associated with belonging to one of the five trajectory groups. Relative to the individuals in group 5 (reference group), group 1 individuals were more likely to be widowed with a relative risk ratio (RRR) of 2.23 (95% confidence interval (CI) = 1.25–3.97), group 2 individuals were more likely to be male (RRR = 1.70; CI = 1.11–2.61) and widowed (RRR = 2.47; CI = 1.16–5.24), group 3 individuals were more likely to be residing in a Tsima intervention village (RRR = 1.89; CI = 1.42–2.52), and group 4 individuals were more likely to be male (RRR = 2.36; CI = 1.47–3.79), aged >30, and with temporary residence status in the study area (RRR = 1.94; CI = 1.11–3.39) (**Table 3**, **Figure 3**; Figure S3 and S4 in the **Online Supplementary Document**).

**Table 3:** Multivariable multinomial regression analyses of factors associated with HIV care retention trajectory groups*

	Group 1: gradually decreasing retention		Group 2: late increasing retention		Group 3: early increasing retention		Group 4: early decreasing retention	
	**RRR (95% CI)**	***P*-value**	**RRR (95% CI)**	***P*-value**	**RRR (95% CI)**	***P*-value**	**RRR (95% CI)**	***P*-value**
**Gender**								
Female	1		1		1		1	
Male	1.20 (0.87–1.66)	0.274	1.70 (1.11–2.61)	0.015	0.77 (0.52–1.13)	0.179	2.36 (1.47–3.79)	<0.001
**Months on ART**								
0–12	1		1		1		1	
13–24	1.20 (0.80–1.80)	0.381	0.77 (0.42–1.42)	0.402	1.14 (0.72–1.80)	0.574	0.98 (0.53–1.80)	0.937
25–36	0.79 (0.52–1.18)	0.245	0.74 (0.42–1.31)	0.302	1.00 (0.65–1.54)	0.999	0.92 (0.52–1.65)	0.788
37–48	1.00 (0.66–1.51)	0.993	0.92 (0.52–1.62)	0.773	1.26 (0.81–1.96)	0.297	0.45 (0.21–0.99)	0.047
49–60	1.16 (0.75–1.81)	0.506	0.70 (0.35–1.39)	0.31	0.67 (0.39–1.16)	0.152	0.58 (0.25–1.34)	0.203
≥61	0.91 (0.62–1.34)	0.643	0.87 (0.51–1.47)	0.593	0.68 (0.43–1.06)	0.085	0.60 (0.31–1.16)	0.128
Age group								
18–29	1		1		1		1	
30–39	0.48 (0.22–1.02)	0.057	0.50 (0.18–1.33)	0.163	0.87 (0.35–2.17)	0.772	0.29 (0.13–0.68)	0.004
40–49	0.44 (0.21–0.95)	0.036	0.40 (0.15–1.07)	0.067	0.72 (0.29–1.78)	0.477	0.10 (0.04–0.25)	<0.001
≥50	0.48 (0.22–1.07)	0.074	0.58 (0.21–1.63)	0.304	0.64 (0.25–1.66)	0.357	0.11 (0.04–0.30)	<0.001
**SES quintiles**								
1	1		1		1		1	
2	0.75 (0.51–1.11)	0.148	0.83 (0.47–1.46)	0.512	0.76 (0.49–1.19)	0.229	0.71 (0.37–1.36)	0.305
3	0.72 (0.48–1.08)	0.116	0.79 (0.43–1.44)	0.442	1.29 (0.84–2.00)	0.249	1.13 (0.61–2.10)	0.697
4	0.71 (0.48–1.06)	0.094	0.89 (0.50–1.59)	0.703	0.89 (0.57–1.40)	0.623	0.76 (0.40–1.47)	0.421
5	0.86 (0.57–1.28)	0.453	1.49 (0.86–2.60)	0.157	0.95 (0.60–1.52)	0.844	1.05 (0.56–1.98)	0.876
**Education**								
Primary	1		1		1		1	
Secondary	1.13 (0.81–1.58)	0.479	1.23 (0.75–2.00)	0.411	0.93 (0.65–1.34)	0.703	1.26 (0.71–2.25)	0.423
Matric & tertiary	1.23 (0.86–1.76)	0.264	1.23 (0.73–2.06)	0.441	0.76 (0.51–1.15)	0.192	0.95 (0.51–1.78)	0.873
Marital status								
Single	1		1		1		1	
Married/cohabiting	1.09 (0.82–1.45)	0.553	1.07 (0.71–1.61)	0.752	1.13 (0.82–1.55)	0.465	0.87 (0.54–1.42)	0.586
Widowed	2.23 (1.25–3.97)	0.007	2.47 (1.16–5.24)	0.019	1.55 (0.79–3.03)	0.202	2.14 (0.78–5.86)	0.139
Separated/divorced	0.74 (0.50-1.11)	0.145	0.77 (0.43-1.39)	0.388	0.69 (0.43-1.10)	0.119	0.83 (0.43-1.61)	0.583
Resident status								
Permanent	1		1		1		1	
Temporary	1.18 (0.80–1.76)	0.408	1.22 (0.72–2.09)	0.46	1.19 (0.76–1.86)	0.451	1.94 (1.11–3.39)	0.02
**Tsima intervention village**								
No	1		1		1		1	
Yes	0.94 (0.74–1.21)	0.647	1.36 (0.95–1.94)	0.095	1.89 (1.42–2.52)	<0.001	1.03 (0.69–1.54)	0.885

ART – antiretroviral therapy, CI – confidence interval, RRR – relative risk ratio, SES – socioeconomic status

*Reference group was Group 5: consistently high retention.

## DISCUSSION

In this study, we identified five distinct trajectories of HIV care retention during 36 months (12 quarters) among people living with HIV and receiving ART in a rural South African region with high HIV prevalence. Although prior research has examined longitudinal trajectories of HIV care retention or ART adherence [28,40-44], this is the first study, to our knowledge, to assess HIV care retention trajectories in Mpumalanga, South Africa, in the context of test-and-treat.

Using GBTM, a novel methodological approach, we showed that patterns of HIV care retention are not uniform in this population. Instead, we identified five distinct retention trajectories, and the factors associated with those groups were strong predictors for following a specific trajectory. We found that 61.4% of the study population had consistently high or increasing retention over time, while 38.6% had decreasing retention. Being female and older (>40 years) was associated with consistently high retention, while being male, younger (18–29 years), and with temporary residence status was associated with early decreasing retention. Education level, SES, and marital status did not independently affect group membership. The number of months on ART had a marginally significant effect on group membership (group 4), however, this is most likely due to the small sample size.

Our study also showed that individuals in the early increasing retention group were more likely to be from villages included in the Tsima study. This is not surprising given the positive impacts of Tsima at the population level. Tsima is the first trial specifically designed to assess whether a community mobilisation intervention addressing barriers to HIV service uptake and promoting treatment as prevention can increase rates of HIV testing, linkage to care, and retention in care. The study showed that testing increased significantly for men and women in intervention communities. The linkage and retention indicators were also slightly higher among intervention communities, with evidence of increased linkage and less default among women in intervention villages. The study also showed that all 90-90-90 indicators were higher in intervention communities at the end of the three-year study. The results of the Tsima trial strengthened the evidence that community mobilisation interventions that address social barriers and focus on treatment as prevention can yield improvements in the HIV care continuum.

Our findings are also in line with other studies, which have shown that retaining young males in HIV care is a challenge [9,10,18,19,21,45-50]. In rural South Africa, migration or mobility for employment, education, and family responsibilities has challenged younger patients’ ability to regularly attend their clinic appointments [51-54]. However, due to migration or mobility, retention in care may be misclassified as not retained or lost to follow-up, when individuals may have remained in care in a clinic in another area [53,55]. Nevertheless, other recent studies in the region have also shown that migration and mobility can lead to men’s lower retention in HIV care due to unexpected work travel and exploitative employer demands. These work demands result in missed appointments and ART interruption because patients cannot plan to have enough medications when they travel, and they are often also denied ART refills at non-home facilities [25,49,50]. Institutional health system discrimination is another important factor that has been shown to affect young men’s retention in HIV care in South Africa in the form of mistreatment by health care providers and lack of confidentiality due to health facility layouts and practices that stigmatise people living with HIV [56]. However, there is still limited evidence regarding the broader impacts of stigma on the HIV care continuum [56,57], and on young men’s HIV care retention in particular.

The finding that older women living with HIV may have high retention could be due to co-morbidities, resulting in increased clinic attendance. Other studies in Mpumalanga have shown that living with HIV is indeed a risk factor for chronic cardiometabolic diseases, and that participation in ART programs in the area has been associated with a greater use of care for chronic conditions [58,59]. Moreover, studies have shown that older people living with HIV in South Africa have greater social support, including higher rates of HIV status disclosure, which can also lead to better ART adherence [44,60,61].

Our study also has limitations that should be highlighted. First, based on survey data collected and analysed by the Tsima parent study, 7% of those in HIV care in the Agincourt HDSS reported receiving treatment at a facility outside the study area. Proportions receiving care elsewhere were equivalent between Tsima intervention and control communities on the survey; thus, bias in comparative findings is unlikely. However, this limitation may lead to an underestimation of HIV care engagement and retention, although it is difficult to estimate how much it might differentially affect the five trajectory patterns. Second, only a few socioeconomic variables were available from the HDSS data source. Variables such as employment status, social support, and distance to the nearest study clinic were unavailable, limiting our ability to characterise socioeconomic predictors of HIV care retention trajectories fully. Third, the trajectory groups we identified may be generalisable to similar rural settings in South Africa, but not to urban settings or other countries. The literature is limited regarding these socioeconomic variables and the urban vs rural comparison, and the findings are mixed. Two studies in KwaZulu-Natal, South Africa found that patients who use public transport to access health facilities were more likely to be in care than those who access clinics on foot [9], and that there are no significant differences in retention between peri-urban and rural clinics [62]. The gaps in the literature highlight a lack of sufficient evidence on interventions targeting men [8], particularly those addressing socioeconomic factors. Further applications of our data-driven approach to population-based cohort data with different and richer covariate information may allow for a more nuanced and in-depth assessment of HIV care retention-associated factors and predictors.

Despite these limitations, however, our study contributes to a better understanding of retention in HIV care in rural South Africa by showing that in this rural community, age, gender, and residence status play an essential role in HIV care retention trajectories, which may be helpful in the design and development of targeted interventions. Furthermore, the results can be used to improve the granularity of HIV epidemic transmission models, which are increasingly used to assess the potential impact of ‘Treatment as Prevention’ [41], and universal test-and-treat [28]. To be useful for policy making, these models should incorporate all features of the HIV care cascade (*i.e.* HIV testing and diagnoses, linkage to care, ART receipt and retention, loss to follow-up, and re-engagement with care). Results from our trajectory analyses can help characterise dynamic rather than binary HIV care retention patterns.

## CONCLUSIONS

In conclusion, GBTM is an innovative and promising approach for a more comprehensive analysis of health and demographic surveillance system (HDSS) data and a better understanding of retention in HIV care. This data-driven analytical approach can identify longitudinal retention trajectories and their associated predictors, and sheds light on the ways HIV care engagement and retention change over time. A better knowledge of the HIV epidemic can support the design of tailored interventions and improve policies on HIV care. Socioeconomic and system-level interventions, particularly those targeting men and addressing issues like expanded differentiated service delivery, streamlined clinic-switching, the ability to obtain ART refills from any site, and psychosocial support, are crucial for improving HIV care retention in high-burden areas.

## Additional material


Online Supplementary Document
