# Supplementary material for: Trajectories and predictors of HIV care retention among individuals receiving ART in rural South Africa: a group-based trajectory modelling analysis
Source: J Glob Health. 2025 Jul 1;15:04187. doi: 10.7189/jogh.15.04187 (PMC12208282; doi:10.7189/jogh.15.04187)
Supplement: Online Supplementary Document [file jogh-15-04187-s001.pdf]

Supplement to: Julien A, Bashingwa JJH, Van Rie A, Dufour MK, Masilela N, West RL, Rebombo D, Gómez-Olivé FX, Kahn K, Lippman SA, Pettifor A, Kabudula CW. Trajectories and predictors of HIV care retention among individuals receiving ART in rural South Africa: a group-based trajectory modelling analysis. J Glob Health. 2025;15:04187.

Figure S1. Rootogram of posterior probabilities

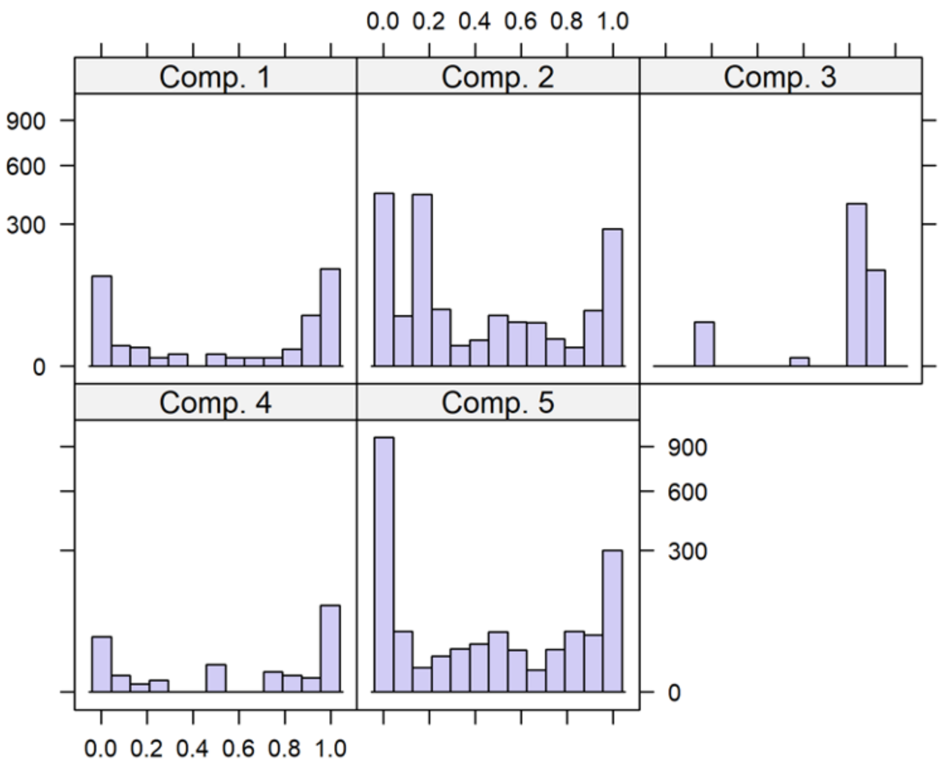

Figure S2. Model Fit Statistics

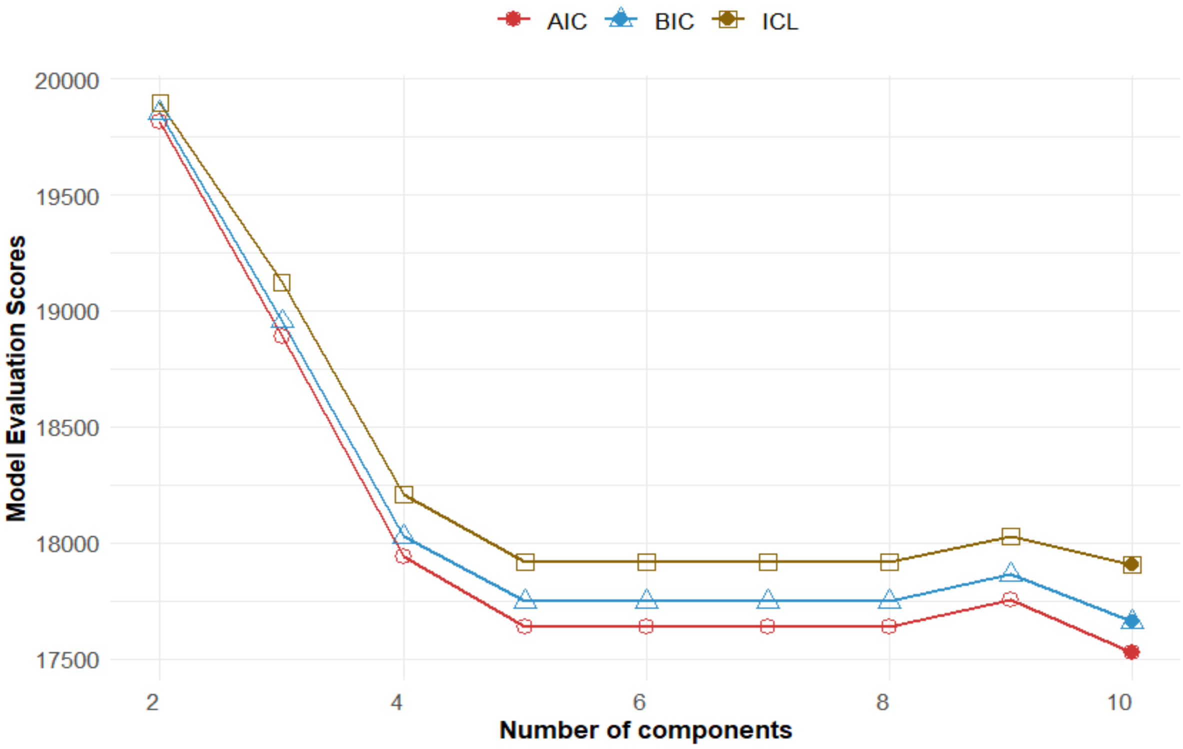

AIC – Akaike information criterion, BIC – Bayesian information criterion,
ICL – integrated classification likelihood

Figure S3. Multivariable multinomial regression analyses of factors associated with HIV care retention trajectory groups (Reference = Group 5: Consistently High Retention)

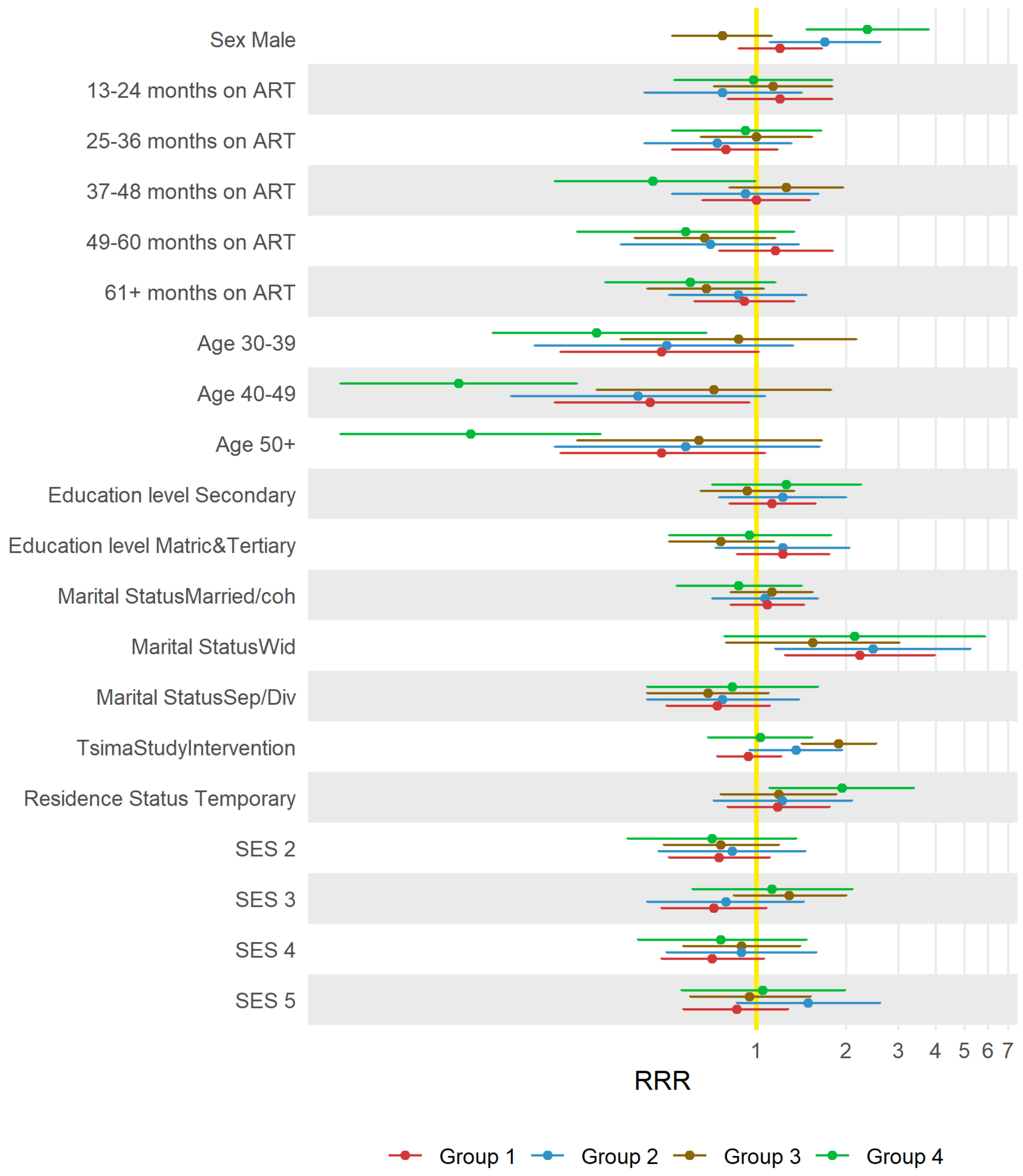

ART – antiretroviral therapy, SES – socioeconomic status, RRR – relative risk ratio

Figure S4. Predicted probabilities of trajectory group membership by retention predictor (Stacked bar visualization)

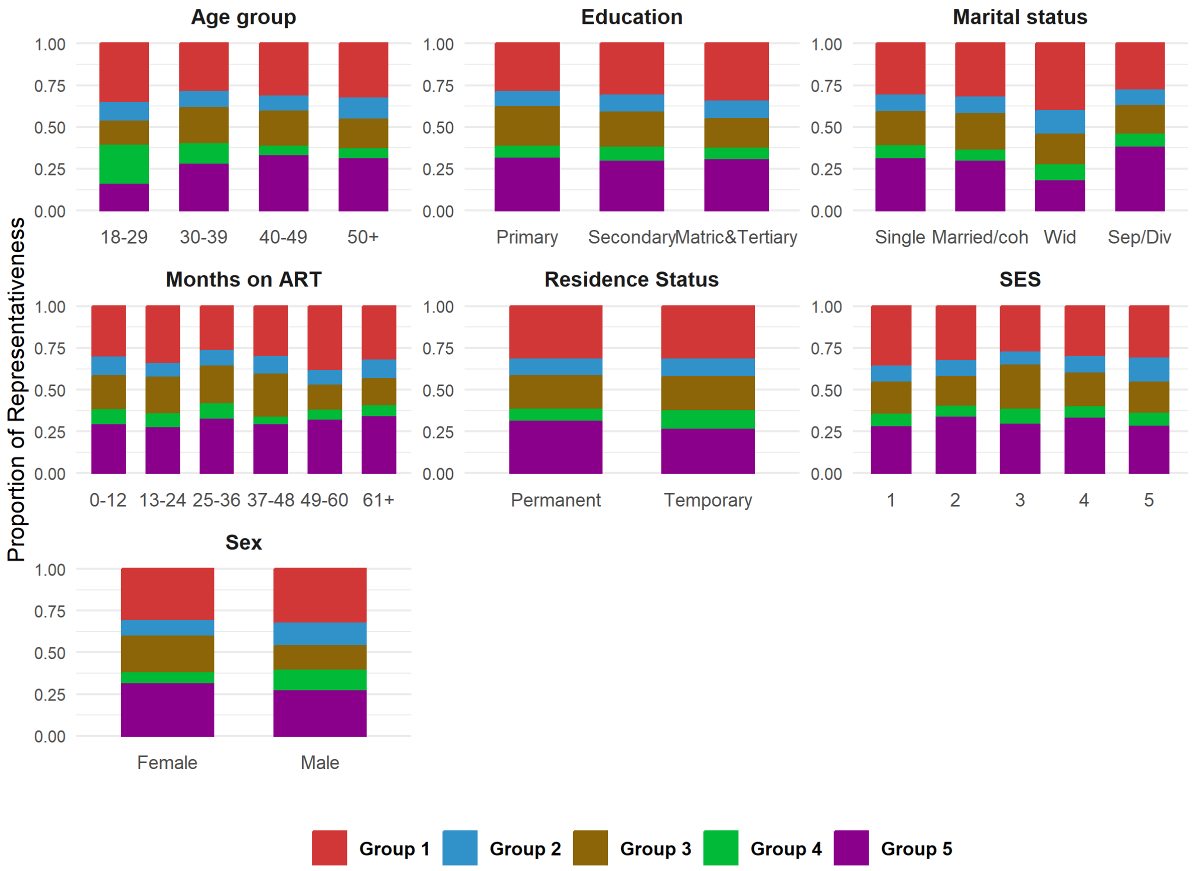

ART – antiretroviral therapy, SES – socioeconomic status

Table S1. Characteristics of participants at baseline (Full Cohort vs. GBTM Cohort)

	**Full Cohort n (%)**	**GBTM Cohort n (%)**	**p-value**
Overall	1839	1689	
Gender			
Female	1448 (78.7%)	1344 (79.6%)	0.57
Male	391 (21.3%)	345 (20.4%)	
Months on ART			
0-12	561 (30.5%)	516 (30.6%)	1
13-24	261 (14.2%)	236 (14.0%)	
25-36	274 (14.9%)	249 (14.7%)	
37-48	258 (14.0%)	238 (14.1%)	
49-60	175 (9.5%)	165 (9.8%)	
61+	310 (16.9%)	285 (16.9%)	
Age Group			
18-29	79 (4.3%)	69 (4.1%)	0.96
30-39	512 (27.8%)	480 (28.4%)	
40-49	836 (45.5%)	770 (45.6%)	
50+	412 (22.4%)	370 (21.9%)	
SES Quintiles			
1	361 (19.6%)	346 (20.5%)	0.99
2	358 (19.5%)	336 (19.9%)	
3	343 (18.7%)	332 (19.7%)	
4	344 (18.7%)	330 (19.5%)	
5	359 (19.5%)	345 (20.4%)	
Missing	74 (4.0%)	0 (0%)	
Education			
Matric&Tertiary	592 (32.2%)	555 (32.9%)	0.96
Primary	394 (21.4%)	364 (21.6%)	
Secondary	813 (44.2%)	770 (45.6%)	
Missing	40 (2.2%)	0 (0%)	
Marital Status			
Married/Cohabiting	610 (33.2%)	575 (34.0%)	0.45
Separated/Divorced	256 (13.9%)	204 (12.1%)	
Single	863 (46.9%)	806 (47.7%)	
Widowed	110 (6.0%)	104 (6.2%)	
Resident Status			
Permanent	1508 (82.0%)	1484 (87.9%)	0.54
Temporary	223 (12.1%)	205 (12.1%)	
Missing	108 (5.9%)	0 (0%)	
Tsima Study Arm			
Control	836 (45.5%)	765 (45.3%)	0.95
Intervention	1003 (54.5%)	924 (54.7%)	

ART – antiretroviral therapy, GBTM – group-based trajectory modelling, SES – socioeconomic status

Table S2. Model fit statistics

Iteration	k	k0	logLik	AIC	BIC	ICL
19	2	2	-9901.93	19813.86	19853.65	19894.11
20	3	3	-9437.4	18890.81	18954.48	19122.93
13	4	4	-8958.61	17939.22	18026.78	18207.54
24	5	5	-8804.55	17637.1	17748.53	17915.94
55	5	6	-8804.55	17637.11	17748.54	17917.48
79	5	7	-8804.55	17637.11	17748.54	17917.46
74	5	8	-8804.55	17637.11	17748.54	17917.45
63	5	9	-8861.96	17751.91	17863.35	18026.11
105	6	10	-8746.12	17526.24	17661.55	17908.12

AIC – Akaike information criterion, BIC – Bayesian information criterion, ICL – integrated classification likelihood, k – components

STROBE Statement—checklist of items that should be included in reports of observational studies

	Item No	Recommendation
**Title and abstract**	1	(*a*) Indicate the study's design with a commonly used term in the title or the abstract
		(*b*) Provide in the abstract an informative and balanced summary of what was done and what was found
**Introduction**		
Background/rationale	2	Explain the scientific background and rationale for the investigation being reported
Objectives	3	State specific objectives, including any prespecified hypotheses
**Methods**		
Study design	4	Present key elements of study design early in the paper
Setting	5	Describe the setting, locations, and relevant dates, including periods of recruitment, exposure, follow-up, and data collection
Participants	6	(*a*) *Cohort study*—Give the eligibility criteria, and the sources and methods of selection of participants. Describe methods of follow-up<br>*Case-control study*—Give the eligibility criteria, and the sources and methods of case ascertainment and control selection. Give the rationale for the choice of cases and controls<br>*Cross-sectional study*—Give the eligibility criteria, and the sources and methods of selection of participants
		(*b*) *Cohort study*—For matched studies, give matching criteria and number of exposed and unexposed<br>*Case-control study*—For matched studies, give matching criteria and the number of controls per case
Variables	7	Clearly define all outcomes, exposures, predictors, potential confounders, and effect modifiers. Give diagnostic criteria, if applicable
Data sources/ measurement	8*	For each variable of interest, give sources of data and details of methods of assessment (measurement). Describe comparability of assessment methods if there is more than one group
Bias	9	Describe any efforts to address potential sources of bias
Study size	10	Explain how the study size was arrived at
Quantitative variables	11	Explain how quantitative variables were handled in the analyses. If applicable, describe which groupings were chosen and why
Statistical methods	12	(*a*) Describe all statistical methods, including those used to control for confounding
		(*b*) Describe any methods used to examine subgroups and interactions
		(*c*) Explain how missing data were addressed
		(*d*) *Cohort study*—If applicable, explain how loss to follow-up was addressed<br>*Case-control study*—If applicable, explain how matching of cases and controls was addressed<br>*Cross-sectional study*—If applicable, describe analytical methods taking account of sampling strategy
		(*e*) Describe any sensitivity analyses

Continued on next page

**Results**		
Participants	13*	(a) Report numbers of individuals at each stage of study—eg numbers potentially eligible, examined for eligibility, confirmed eligible, included in the study, completing follow-up, and analysed
		(b) Give reasons for non-participation at each stage
		(c) Consider use of a flow diagram
Descriptive data	14*	(a) Give characteristics of study participants (eg demographic, clinical, social) and information on exposures and potential confounders
		(b) Indicate number of participants with missing data for each variable of interest
		(c) *Cohort study*—Summarise follow-up time (eg, average and total amount)
Outcome data	15*	*Cohort study*—Report numbers of outcome events or summary measures over time
		Case-control study—Report numbers in each exposure category, or summary measures of exposure
		Cross-sectional study—Report numbers of outcome events or summary measures
Main results	16	(*a*) Give unadjusted estimates and, if applicable, confounder-adjusted estimates and their precision (eg, 95% confidence interval). Make clear which confounders were adjusted for and why they were included
		(*b*) Report category boundaries when continuous variables were categorized
		(*c*) If relevant, consider translating estimates of relative risk into absolute risk for a meaningful time period
Other analyses	17	Report other analyses done—eg analyses of subgroups and interactions, and sensitivity analyses
**Discussion**		
Key results	18	Summarise key results with reference to study objectives
Limitations	19	Discuss limitations of the study, taking into account sources of potential bias or imprecision. Discuss both direction and magnitude of any potential bias
Interpretation	20	Give a cautious overall interpretation of results considering objectives, limitations, multiplicity of analyses, results from similar studies, and other relevant evidence
Generalisability	21	Discuss the generalisability (external validity) of the study results
**Other information**		
Funding	22	Give the source of funding and the role of the funders for the present study and, if applicable, for the original study on which the present article is based

*Give information separately for cases and controls in case-control studies and, if applicable, for exposed and unexposed groups in cohort and cross-sectional studies.

Note: An Explanation and Elaboration article discusses each checklist item and gives methodological background and published examples of transparent reporting. The STROBE checklist is best used in conjunction with this article (freely available on the Web sites of PLoS Medicine at http://www.plosmedicine.org/, Annals of Internal Medicine at http://www.annals.org/, and Epidemiology at http://www.epidem.com/). Information on the STROBE Initiative is available at www.strobe-statement.org.
